# Roles of the m6A methyltransferases METTL3, METTL14, and WTAP in pulmonary tuberculosis

**DOI:** 10.3389/fimmu.2022.992628

**Published:** 2022-12-07

**Authors:** Tian-Ping Zhang, Rui Li, Li-Jun Wang, Qian Huang, Hong-Miao Li

**Affiliations:** ^1^ Department of Rheumatology and Immunology, The First Affiliated Hospital of USTC, Division of Life Sciences and Medicine, University of Science and Technology of China, Hefei, China; ^2^ Department of Nosocomial Infection Management, The First Affiliated Hospital of Anhui Medical University, Hefei, China; ^3^ Department of Infectious Diseases, The First Affiliated Hospital of Anhui Medical University, Hefei, China; ^4^ Department of Public Health, Medical Department, Qinghai University, Xining, China; ^5^ Department of Epidemiology and Biostatistics, School of Public Health, Anhui Medical University, Hefei, China

**Keywords:** m6A methyltransferase, single nucleotide polymorphisms, pulmonary tuberculosis, METTL3, METTL14, WTAP

## Abstract

**Objective:**

The aim of the current study was to investigate the contributing role of gene variation and transcription levels among the m6A methyltransferases METTL3, METTL14, and WTAP in pulmonary tuberculosis (PTB).

**Methods:**

A case-control study including 461 PTB patients and 467 normal controls was designed for genotyping. Three SNPs in *METTL3* (rs1061027, rs1139130, rs1061026), three SNPs in *METTL14* (rs62328061, rs4834698, rs1064034), and two SNPs in *WTAP* (rs1853259, rs11752345) were genotyped *via* the SNPscan™ technique. *METTL3*, *METTL14*, and *WTAP* transcription levels were determined in 78 PTB patients and 86 controls *via* quantitative real-time reverse-transcription PCR.

**Results:**

Frequencies of the *METTL14* rs62328061 GG genotype, *WTAP* rs11752345 CT genotype, and T allele were significantly increased in PTB patients compared to controls. An increased risk of rs62328061 was detected in a recessive model, and a decreased risk of rs11752345 was detected in a dominant model in the PTB group. *METTL3* gene variation was not associated with PTB risk. The *METTL3* rs1139130 GG genotype was significantly increased with drug resistance, and the G allele was significantly decreased with drug-induced liver injury in PTB patients. A reduced frequency of the *METTL14* rs62328061 G allele was associated with leukopenia, a reduced frequency of the *WTAP* rs11752345 T allele was associated with sputum smear positivity, and a higher frequency of the *METTL14* rs4834698 TC genotype was evident in PTB patients with hypoproteinemia. Compared to controls, *METTL3*, *METTL14*, and *WTAP* transcription levels in PTB patients were significantly decreased, and the level of WTAP was increased in PTB patients with drug resistance. METTL3 level was negatively associated with erythrocyte sedimentation rate and aspartate aminotransferase, and METTL14 level was negatively correlated with alanine aminotransferase and aspartate aminotransferase.

**Conclusion:**

*METTL14* rs62328061 and *WTAP* rs11752345 variants were associated with the genetic background of PTB, and METTL3, METTL14, and WTAP levels were abnormally decreased, suggesting that these m6A methyltransferases may play important roles in PTB.

## Introduction

Tuberculosis (TB) is a common infectious disease caused by *Mycobacterium tuberculosis* (MTB), and it remains one of the most serious public health problems worldwide (1). A World Health Organization update reported that there was an estimated 9.9 million new incident TB patients globally in 2021 (2). It is well known that people infected with MTB can develop several possible outcomes, such as MTB clearance, primary TB, latent TB infection, and active TB. Studies indicate that approximately 10% of individuals infected with MTB will eventually progress to active TB (3, 4). The occurrence and development of TB is mainly influenced by complex interactions between the MTB strain, the external environment, and genetic factors (5, 6). Host genetics are the essential factor determining disease susceptibility and outcomes after MTB infection in many case-control studies, animal model studies, and twin and family studies (7, 8). Continuing studies on the roles of genetic variants in TB susceptibility would contribute to the development of advantageous approaches to TB prevention, diagnosis, and treatment, and many genetic variations have been shown to be associated with susceptibility to TB (9, 10).

N6-methyladenosine (m6A) is formed *via* methylation of the sixth N atom on the adenine base, which is reportedly the most abundant post-transcriptional modification of RNA in eukaryotes, particularly messenger RNA (mRNA) (11). m6A regulates post-transcriptional mRNA levels in a dynamic and reversible manner (12). m6A modification is regulated by several key regulators, including RNA methyltransferases (METTL3, METTL14, and WTAP), demethylases, and m6A-binding proteins (13). m6A modification is involved in many important biological processes, and it is closely related to the occurrence and development of many diseases.

Epigenetic modification, including DNA methylation and noncoding RNAs, reportedly contributes to pulmonary TB (PTB) progression (14, 15). In recent years m6A methylation was also found in the *MTB* genome (16). Therefore, m6A methylation may be involved in the pathogenesis of PTB. Strong evidence indicates that abnormal expression of m6A key regulators may result in abnormal RNA m6A modification, leading to a variety of diseases (17, 18). In addition, some functional single nucleotide polymorphisms (SNPs) in specific regions of m6A key regulator genes may influence m6A methylation, thereby affecting disease development. For example, several SNPs in *METTL3* and *METTL14* genes have been associated with neuroblastoma, Wilms’ tumor, acute lymphoblastic leukemia, and autoimmune thyroid disease (19–22). In a previous study, genetic variations in m6A demethylases FTO could affect susceptibility to PTB (23), but few studies have investigated associations between variation in key m6A modification regulatory genes and PTB risk. The current epidemiological study was conducted to identify novel m6A methyltransferases SNPs associated with susceptibility to PTB. The study assessed associations between three m6A methyltransferase genes (*METTL3*, *METTL14*, and *WTAP*) SNPs, as well as their transcription levels, and PTB risk.

## Materials and methods

### Study subjects

In this case-control study, 461 PTB patients and 467 normal controls were consecutively enrolled, and associations between m6A methyltransferase gene polymorphisms and PTB susceptibility were analyzed. Then, 78 PTB patients and 86 normal controls were enrolled to detect m6A methyltransferase transcription levels. All PTB patients were recruited from the Department of Tuberculosis at Anhui Chest Hospital, whereas normal controls were selected from a health center in the same area. PTB patients were diagnosed by specialists based on indicative clinical symptoms, chest radiography, sputum and/or bronchoalveolar lavage fluid MTB culture, microscopy for acid fast bacilli, and effects of anti-TB treatment. The exclusion criteria included HIV positivity, hepatitis, malignancy, and immunodeficiency. Individuals without a history of TB, malignant tumor, HIV or other infectious diseases were included in the study as normal controls. The control group was required to be asymptomatic with negative sputum smears and cultures, and normal chest radiographs. All subjects were of Chinese Han ethnicity.

The study was approved by the Medical Ethics Committee of Anhui Medical University (approval number 20200250). After obtaining informed consent, peripheral blood samples and relevant information were collected from each participant with the help of professional physicians. The data collected included basic demographic characteristics and some clinical data such as fever, drug resistance, drug-induced liver injury (DILI), pulmonary infection, leukopenia, sputum smears, total bilirubin, aspartate aminotransferase (AST), alanine aminotransferase (ALT), and erythrocyte sedimentation rate (ESR).

### SNP selection

The three m6A methyltransferases METTL3, METTL14, and WTAP were included in the analyses, and the functional SNPs in their corresponding genes were chosen based on previous studies (24). Existing studies investigating associations between *METTL3*, *METTL14*, and *WTAP* gene polymorphisms and human diseases were systematically reviewed, and the SNPs related to human diseases were identified. Ensembl Genome Browser 85 and CHBS_1000g software were then used to obtain genotype data on these genes derived from Han Chinese people living in Beijing, and the tag SNPs of these genes were selected *via* the pairwise option of HaploView 4.0 software (Cambridge, MA, USA). The tag SNP selection criteria were: (1) a minor allele frequency ≥ 5% in CHB_1000g, and an *r* ^2^ threshold > 0.8; (2) the common SNPs located within the chromosome locus transcribed into *METTL3*, *METTL14*, or *WTAP* and their flanking 2000 bp regions were captured; (3) potentially functional SNPs located within the 5’ untranslated region, 3’ untranslated region, and exon were preferentially selected. Lastly, three SNPs in the *METTL3* gene (rs1061027, rs1139130, rs1061026), three SNPs in the *METTL14* gene (rs62328061, rs4834698, rs1064034), and two SNPs in the *WTAP* gene (rs1853259, rs11752345) were selected.

### DNA extraction and genotyping

Samples of approximately 5 mL of peripheral early morning fasting blood were collected from all subjects, and genomic DNA was extracted *via* a Flexi Gene-DNA Kit (Qiagen, Valencia, CA) for genotyping. Genotyping of all DNA samples for the selected SNPs was conducted *via* the Three SNPs in METTL3 (rs1061027, rs1139130, rs1061026), three SNPs in METTL14 (rs62328061, rs4834698, rs1064034), and two SNPs in WTAP (rs1853259, rs11752345) were genotyped *via* the SNPscan™ technique, with technical support from the Center for Genetic and Genomic Analysis, Genesky Biotechnologies Inc. (Shanghai). Only participants with 100% genotyping success for the selected SNPs were included in the final analysis.

### Quantitative real-time reverse-transcription PCR

Peripheral blood mononuclear cells (PBMCs) were isolated from 3 mL anticoagulant peripheral blood, and total RNA was extracted using TRIzol Reagent (Invitrogen, Carlsbad, CA, USA). A NanoDrop 2000 spectrophotometer (Thermo Scientific, USA) was used to determine total RNA concentrations. Total RNA was then reverse transcribed into cDNA by the PrimeScript™ RT Reagent Kit (Takara Bio Inc., Japan). In this experiment a 20-μL reverse transcription reaction system with a maximum of 1 μg total RNA was used, and the amount of total RNA needed in the reaction system was determined based on the RNA concentration.

METTL3, METTL14, and WTAP mRNA levels in PBMCs were detected *via* quantitative real-time reverse transcription (qRT) PCR with SYBR Green (SYBR Premix Ex Taq II, Takara Bio Inc., Japan). qRT-PCRs were conducted using a QuantStudio 12K Flex Real-Time PCR System (Applied Biosystems, Foster City, CA, USA), and the cycle conditions of reactions were 95°C for 1 min, followed by 42 cycles at 95°C for 10 sec, 60°C for 30 sec, and 72°C for 1 min. Relative transcription levels of METTL3, METTL14, and WTAP were calculated *via* comparisons with the housekeeping gene (internal control) β-actin in the same sample, and the 2^-△△Ct^ method was used to express levels (25).

### Statistical analysis

Whether the genotypes distribution of all SNPs in normal controls were in Hardy-Weinberg equilibrium was assessed *via* the Chi-square test. Associations between m6A methyltransferase gene polymorphisms and PTB susceptibility were estimated *via* odds ratios (*OR*) and 95% confidence intervals (*CI*) with logistic regression analyses. Two genetic models (dominant and recessive) were used to analyze associations between these SNPs and PTB risk, and haplotype analysis was conducted *via* SHEsis software (26). Transcription levels of *METTL3*, *METTL14*, and *WTAP* are expressed as median and quartile intervals, and the Mann-Whitney *U* test and the Kruskal-Wallis *H* test were respectively used to evaluate differences in these m6A methyltransferase levels between two groups and among three groups. Correlations between these m6A methyltransferase levels and experimental indexes in PTB patients were analyzed *via* the Spearman rank correlation coefficient test. All statistical analyses were conducted with SPSS 23.0 (SPSS Inc., IL, USA), and two-sided *p* values of < 0.05 were deemed to indicate statistical significance.

## Results

### Associations between m6A methyltransferase gene polymorphisms and PTB susceptibility

The PTB group genotyped included 194 females and 267 males, with a mean age of 45.56 ± 17.75 years. The normal control group genotyped included 265 females and 202 males, with a mean age of 43.38 ± 13.86 years. The genotype distributions of all SNPs in normal controls were in Hardy-Weinberg equilibrium, and the allele and genotype frequencies of these SNPs are shown in **Table 1**.

**Table 1:** Association between *METTL3*, *METTL14*, and *WTAP* genes polymorphism and PTB risk.

SNP	Analyze model		PTB patients	Controls	*P* value	*OR* (95% *CI*)
*METTL3*						
rs1139130	Genotype	GG	52 (11.28)	65 (13.92)	0.327	1.065 (0.808,1.403)
		AG	217 (47.07)	207 (44.33)	0.656	0.813 (0.536,1.231)
		AA	192 (41.65)	195 (41.76)	Reference	
	Allele	G	321 (34.82)	337 (36.08)	0.599	1.020 (0.953,1.091)
		A	601 (65.18)	597 (63.92)	Reference	
	Dominant model	AA	192 (41.65)	195 (41.76)	0.974	0.997 (0.857,1.161)
		AG+GG	269 (58.35)	272 (58.24)	Reference	
	Recessive model	GG	52 (11.28)	65 (13.92)	0.226	1.031 (0.981,1.082)
		AG+AA	409 (88.72)	402 (86.08)	Reference	
rs1061026	Genotype	GG	4 (0.87)	3 (0.64)	0.723	1.313 (0.292,5.905)
		TG	68 (14.75)	81 (17.34)	0.289	0.829 (0.581,1.175)
		TT	389 (84.38)	383 (82.01)	Reference	
	Allele	G	76 (8.24)	87 (9.31)	0.415	1.012 (0.984,1.041)
		T	846 (91.76)	847 (90.69)	Reference	
	Dominant model	TT	389 (84.38)	383 (82.01)	0.335	0.868 (0.651,1.157)
		TG+GG	72 (15.62)	84 (17.99)	Reference	
	Recessive model	GG	4 (0.87)	3 (0.64)	0.692	1.351 (0.304,6.002)
		TG+TT	457 (99.13)	464 (99.36)	Reference	
rs1061027	Genotype	AA	15 (3.25)	21 (4.50)	0.328	0.712 (0.360,1.407)
		CA	142 (30.80)	143 (30.62)	0.943	0.990 (0.747,1.311)
		CC	304 (65.94)	303 (64.88)	Reference	
	Allele	C	172 (18.66)	185 (19.81)	0.529	1.014 (0.970,1.060)
		A	750 (81.34)	749 (80.19)	Reference	
	Dominant model	CC	304 (65.94)	303 (64.88)	0.734	1.016 (0.926,1.116)
		CA+AA	157 (34.06)	164 (35.12)	Reference	
	Recessive model	AA	15 (3.25)	21 (4.50)	0.327	0.724 (0.378,1.386)
		CA+CC	446 (96.75)	446 (95.50)	Reference	
*METTL14*						
rs1064034	Genotype	AA	41 (8.89)	37 (7.92)	0.858	1.045 (0.645,1.692)
		AT	192 (41.65)	215 (46.04)	0.211	0.842 (0.643,1.102)
		TT	228 (49.46)	215 (46.04)	Reference	
	Allele	A	274 (29.72)	289 (30.94)	0.566	1.018 (0.958,1.081)
		T	648 (70.28)	645 (69.06)	Reference	
	Dominant model	TT	228 (49.46)	215 (46.04)	0.297	0.937 (0.828,1.059)
		AT+AA	233 (50.54)	252 (53.96)	Reference	
	Recessive model	AA	41 (8.89)	37 (7.92)	0.594	1.123 (0.734,1.718)
		AT+TT	420 (91.11)	430 (92.08)	Reference	
rs62328061	Genotype	GG	18 (3.90)	7 (1.50)	**0.043**	2.501 (1.031,6.069)
		AG	114 (24.73)	140 (29.98)	0.117	0.792 (0.592,1.060)
		AA	329 (71.37)	320 (68.52)	Reference	
	Allele	G	150 (16.27)	154 (16.49)	0.898	1.003 (0.963,1.044)
		A	772 (83.73)	780 (83.51)	Reference	
	Dominant model	AA	329 (71.37)	320 (68.52)	0.345	0.910 (0.747,1.107)
		AG+GG	132 (28.63)	147 (31.48)	Reference	
	Recessive model	GG	18 (3.90)	7 (1.50)	**0.024**	2.605 (1.098,6.177)
		AG+AA	443 (96.10)	460 (98.50)	Reference	
rs4834698	Genotype	CC	108 (23.43)	97 (20.77)	0.786	1.053 (0.727,1.524)
		TC	225 (48.81)	249 (53.32)	0.314	0.854 (0.628,1.161)
		TT	128 (27.77)	121 (25.91)	Reference	
	Allele	C	441 (47.83)	443 (47.43)	0.863	1.008 (0.917,1.109)
		T	481 (52.17)	491 (52.57)	Reference	
	Dominant model	TT	128 (27.77)	121 (25.91)	0.524	0.975 (0.902,1.054)
		TC +CC	333 (72.23)	346 (74.09)	Reference	
	Recessive model	CC	108 (23.43)	97 (20.77)	0.329	1.128 (0.885,1.437)
		TC +TT	353 (76.57)	370 (79.23)	Reference	
*WTAP*						
rs11752345	Genotype	TT	1 (0.22)	4 (0.86)	0.246	0.273 (0.030,2.449)
		CT	83 (18.00)	52 (11.13)	**0.004**	1.740 (1.120,2.529)
		CC	377 (81.78)	411 (88.01)	Reference	
	Allele	T	85 (9.22)	60 (6.42)	**0.025**	0.970 (0.945,0.996)
		C	837 (90.78)	874 (93.58)	Reference	
	Dominant model	CC	377 (81.78)	411 (88.01)	**0.008**	1.520 (1.112,2.077)
		CT+TT	84 (18.22)	56 (11.99)	Reference	
	Recessive model	TT	1 (0.22)	4 (0.86)	0.183	0.253 (0.028,2.257)
		CT+CC	460 (99.78)	463 (99.14)	Reference	
rs1853259	Genotype	GG	63 (13.67)	74 (15.85)	0.497	0.872 (0.586,1.297)
		AG	229 (49.67)	220 (47.11)	0.658	1.066 (0.804,1.412)
		AA	169 (36.66)	173 (37.04)	Reference	
	Allele	G	355 (38.5)	368 (39.4)	0.692	0.977 (0.799,1.161)
		A	567 (61.5)	566 (60.6)	Reference	
	Dominant model	AA	169 (36.66)	173 (37.04)	0.903	1.006 (0.912,1.110)
		AG+GG	292 (63.34)	294 (62.96)	Reference	
	Recessive model	GG	292 (13.67)	294 (15.85)	0.858	0.989 (0.875,1.118)
		AG+GG	398 (86.33)	393 (84.15)	Reference	

Bold value means P < 0.05.

In the *METTL14* gene, the frequency of the rs62328061 GG genotype was significantly higher in PTB patients than in normal controls, and the genotype was associated with PTB susceptibility in the recessive model (GG versus AA *p* = 0.043; GG versus AG+AA *p* = 0.024). There was no significant association between the rs1064034 variant and PTB risk. In comparisons of *WTAP* rs11752345 variant genotype and allele frequencies between the PTB group and control group, the CT genotype and T allele frequencies were significantly increased in PTB patients (CT versus CC *p* = 0.004; T versus C *p* = 0.025). There was a decreased risk of the rs11752345 variant in the dominant model (CC versus CT+TT *p* = 0.008). PTB risk was not significantly associated with the *WTAP* rs1853259 variant, or with the *METTL3* rs1139130, rs1061026, or rs1061027 variants (all *p* > 0.05).

A case-only analysis was performed to analyze associations between *METTL3*, *METTL14*, and *WTAP* gene variants and common clinical features of PTB (**Table 2**). *METTL3* gene rs1139130 GG genotype frequency was significantly positively associated with drug resistance (*p* = 0.017), whereas rs1139130 G allele frequency was significantly negatively associated with DILI (*p* = 0.041) in PTB patients. The *METTL14* gene G allele frequency of the rs62328061 variant was significantly associated with a decreased risk of leukopenia (*p* = 0.016), and a higher frequency of the rs4834698 TC genotype was observed in PTB patients with hypoproteinemia (*p* = 0.042). A decreased frequency of the *WTAP* rs11752345 T allele was significantly associated with sputum smear positivity (*p* = 0.041). There were no significant associations between other SNPs and the clinical features of PTB.

**Table 2:** Associations between *METTL3*, *METTL14*, and *WTAP* genes polymorphisms and clinical features of PTB patients.

SNP	Allele	Clinical features	Group	Genotype n (%)			*P* value	Allele n (%)		*P* value
	(M/m)			MM	Mm	mm		M	m	
*METTL3*										
rs1139130	A/G	fever	+	37 (51.39)	27 (37.5)	8 (11.11)	0.165	101 (70.14)	43 (29.86)	0.174
			–	155 (39.85)	190 (48.84)	44 (11.31)		500 (64.27)	278 (35.73)	
		drug resistance	+	32 (40.51)	30 (37.97)	17 (21.52)	**0.017**	94 (59.49)	64 (40.51)	0.114
			–	195 (42.39)	217 (47.17)	48 (10.43)		607 (65.98)	313 (34.02)	
		DILI	+	39 (51.32)	32 (42.11)	5 (6.58)	0.119	110 (72.37)	42 (27.63)	**0.041**
			–	188 (40.6)	215 (46.44)	60 (12.96)		591 (63.82)	335 (36.18)	
		pulmonary infection	+	45 (48.39)	37 (39.78)	11 (11.83)	0.377	127 (68.28)	59 (31.72)	0.307
			–	182 (40.81)	210 (47.09)	54 (12.11)		574 (64.35)	318 (35.65)	
		hypoproteinemia	+	30 (53.57)	20 (35.71)	6 (10.71)	0.179	80 (71.43)	32 (28.57)	0.133
			–	197 (40.79)	227 (47)	59 (12.22)		621 (64.29)	345 (35.71)	
		leukopenia	+	15 (41.67)	18 (50.00)	3 (8.33)	0.744	48 (66.67)	24 (33.33)	0.763
			–	212 (42.15)	229 (45.53)	62 (12.33)		653 (64.91)	353 (35.09)	
		sputum smear-positive	+	60 (39.22)	72 (47.06)	21 (13.73)	0.69	192 (62.75)	114 (37.25)	0.390
			–	149 (42.94)	157 (45.24)	41 (11.82)		455 (65.56)	239 (34.44)	
rs1061026	T/G	fever	+	61 (84.72)	10 (13.89)	1 (1.39)	0.856	132 (91.67)	12 (8.33)	0.966
			–	328 (84.32)	58 (14.91)	3 (0.77)		714 (91.77)	64 (8.23)	
		drug resistance	+	67 (84.81)	11 (13.92)	1 (1.27)	0.786	145 (91.77)	13 (8.23)	0.917
			–	385 (83.7)	72 (15.65)	3 (0.65)		842 (91.52)	78 (8.48)	
		DILI	+	69 (90.79)	7 (9.21)	0 (0)	0.186	145 (95.39)	7 (4.61)	0.066
			–	383 (82.72)	76 (16.41)	4 (0.86)		842 (90.93)	84 (9.07)	
		pulmonary infection	+	83 (89.25)	10 (10.75)	0 (0)	0.247	176 (94.62)	10 (5.38)	0.098
			–	369 (82.74)	73 (16.37)	4 (0.90)		811 (90.92)	81 (9.08)	
		hypoproteinemia	+	46 (82.14)	10 (17.86)	0 (0)	0.694	102 (91.07)	10 (8.93)	0.845
			–	406 (84.06)	73 (15.11)	4 (0.83)		885 (91.61)	81 (8.39)	
		leukopenia	+	32 (88.89)	4 (11.11)	0 (0)	0.649	68 (94.44)	4 (5.56)	0.362
			–	420 (83.50)	79 (15.71)	4 (0.80)		919 (91.35)	87 (8.65)	
		sputum smear-positive	+	131 (85.62)	21 (13.73)	1 (0.65)	0.743	283 (92.48)	23 (7.52)	0.504
			–	288 (83.00)	57 (16.43)	2 (0.58)		633 (91.21)	61 (8.79)	
rs1061027	C/A	fever	+	46 (63.89)	23 (31.94)	3 (4.17)	0.857	115 (79.86)	29 (20.14)	0.619
			–	258 (66.32)	119 (30.59)	12 (3.08)		635 (81.62)	143 (18.38)	
		drug resistance	+	48 (60.76)	25 (31.65)	6 (7.59)	0.066	121 (76.58)	37 (23.42)	0.095
			–	308 (66.96)	140 (30.43)	12 (2.61)		756 (82.17)	164 (17.83)	
		DILI	+	47 (61.84)	27 (35.53)	2 (2.63)	0.586	121 (79.61)	31 (20.39)	0.550
			–	309 (66.74)	138 (29.81)	16 (3.46)		756 (81.64)	170 (18.36)	
		pulmonary infection	+	61 (65.59)	27 (29.03)	5 (5.38)	0.474	149 (80.11)	37 (19.89)	0.631
			–	295 (66.14)	138 (30.94)	13 (2.91)		728 (81.61)	164 (18.39)	
		hypoproteinemia	+	41 (73.21)	14 (25.00)	1 (1.79)	0.454	96 (85.71)	16 (14.29)	0.211
			–	315 (65.22)	151 (31.26)	17 (3.52)		781 (80.85)	185 (19.15)	
		leukopenia	+	22 (61.11)	13 (36.11)	1 (2.78)	0.756	57 (79.17)	15 (20.83)	0.622
			–	334 (66.4)	152 (30.22)	17 (3.38)		820 (81.51)	186 (18.49)	
		sputum smear-positive	+	95 (62.09)	52 (33.99)	6 (3.92)	0.548	242 (79.08)	64 (20.92)	0.305
			–	233 (67.15)	102 (29.39)	12 (3.46)		568 (81.84)	126 (18.16)	
*METTL14*										
rs1064034	T/A	fever	+	37 (51.39)	33 (45.83)	2 (2.78)	0.135	107 (74.31)	37 (25.69)	0.250
			–	191 (49.10)	159 (40.87)	39 (10.03)		541 (69.54)	237 (30.46)	
		drug resistance	+	46 (58.23)	26 (32.91)	7 (8.86)	0.295	118 (74.68)	40 (25.32)	0.301
			–	228 (49.57)	194 (42.17)	38 (8.26)		650 (70.65)	270 (29.35)	
		DILI	+	40 (52.63)	31 (40.79)	5 (6.58)	0.824	111 (73.03)	41 (26.97)	0.600
			–	234 (50.54)	189 (40.82)	40 (8.64)		657 (70.95)	269 (29.05)	
		pulmonary infection	+	45 (48.39)	42 (45.16)	6 (6.45)	0.566	132 (70.97)	54 (29.030)	0.927
			–	229 (51.35)	178 (39.91)	39 (8.74)		636 (71.30)	256 (28.70)	
		hypoproteinemia	+	32 (57.14)	21 (37.50)	3 (5.36)	0.515	95 (71.97)	37 (28.03)	0.764
			–	242 (50.10)	199 (41.20)	42 (8.70)		683 (70.70)	283 (29.30)	
		leukopenia	+	23 (63.89)	12 (33.33)	1 (2.78)	0.196	58 (80.56)	14 (19.44)	0.071
			–	251 (49.90)	208 (41.35)	44 (8.75)		710 (70.58)	296 (29.42)	
		sputum smear-positive	+	82 (53.59)	55 (35.95)	16 (10.46)	0.301	219 (71.57)	87 (28.43)	0.828
			–	172 (49.57)	148 (42.65)	27 (7.78)		492 (70.89)	202 (29.11)	
rs62328061	A/G	fever	+	51 (70.83)	20 (27.78)	1 (1.39)	0.426	122 (84.72)	22 (15.28)	0.726
			–	278 (71.47)	94 (24.16)	17 (4.37)		650 (83.55)	128 (16.45)	
		drug resistance	+	58 (73.42)	19 (24.05)	2 (2.53)	0.906	135 (85.44)	23 (14.56)	0.725
			–	332 (72.17)	112 (24.35)	16 (3.48)		776 (84.35)	144 (15.65)	
		DILI	+	55 (72.37)	19 (25.00)	2 (2.63)	0.928	129 (84.87)	23 (15.13)	0.895
			–	335 (72.35)	112 (24.19)	16 (3.46)		782 (84.45)	144 (15.55)	
		pulmonary infection	+	68 (73.12)	23 (24.73)	2 (2.15)	0.781	159 (85.48)	27 (14.52)	0.686
			–	322 (72.2)	108 (24.22)	16 (3.59)		752 (84.03)	140 (15.7)	
		hypoproteinemia	+	38 (67.86)	16 (28.57)	2 (3.57)	0.721	92 (82.14)	20 (17.86)	0.465
			–	352 (72.88)	115 (23.81)	16 (3.31)		819 (84.78)	147 (15.22)	
		leukopenia	+	32 (88.89)	4 (11.11)	0 (0)	0.064	68 (94.44)	4 (5.56)	**0.016**
			–	358 (71.17)	127 (25.25)	18 (3.58)		843 (83.80)	163 (16.20)	
		sputum smear-positive	+	109 (71.24)	37 (24.18)	7 (4.58)	0.731	255 (83.33)	51 (16.67)	0.537
			–	253 (72.91)	83 (23.92)	11 (3.17)		589 (84.87)	105 (15.13)	
rs4834698	T/C	fever	+	15 (20.83)	40 (55.56)	17 (23.61)	0.321	70 (48.61)	74 (51.39)	0.352
			–	113 (29.05)	185 (47.56)	91 (23.39)		411 (52.83)	367 (47.17)	
		drug resistance	+	19 (25.68)	35 (47.30)	20 (27.03)	0.716	73 (49.32)	75 (50.68)	0.450
			–	109 (28.17)	190 (49.10)	88 (22.74)		408 (52.71)	366 (47.29)	
		DILI	+	20 (29.41)	36 (52.94)	12 (17.65)	0.474	76 (55.88)	60 (44.12)	0.348
			–	108 (27.48)	189 (48.09)	96 (24.43)		405 (51.53)	381 (48.47)	
		pulmonary infection	+	18 (21.95)	43 (52.44)	21 (25.61)	0.430	79 (48.17)	85 (51.83)	0.258
			–	110 (29.02)	182 (48.02)	87 (22.96)		402 (53.03)	356 (46.97)	
		hypoproteinemia	+	6 (15.00)	27 (67.50)	7 (17.50)	**0.042**	39 (48.75)	41 (51.25)	0.522
			–	122 (28.98)	198 (47.03)	101 (23.99)		442 (52.49)	400 (47.51)	
		leukopenia	+	6 (20.00)	13 (43.33)	11 (36.67)	0.193	25 (41.67)	35 (58.33)	0.092
			–	122 (28.31)	212 (49.19)	97 (22.51)		456 (52.90)	406 (47.10)	
		sputum smear-positive	+	34 (26.98)	65 (51.59)	27 (21.43)	0.933	133 (52.78)	119 (47.22)	0.948
			–	82 (27.70)	147 (49.66)	67 (22.64)		311 (52.53)	281 (47.47)	
*WTAP*										
rs11752345	C/T	fever	+	55 (76.39)	17 (23.61)	0 (0)	0.372	127 (88.19)	17 (11.81)	0.243
			–	322 (82.78)	66 (16.97)	1 (0.26)		710 (91.26)	68 (8.74)	
		drug resistance	+	65 (82.28)	14 (17.72)	0 (0)	0.832	144 (91.14)	14 (8.86)	0.983
			–	380 (82.61)	78 (16.96)	2 (0.43)		838 (91.09)	82 (8.91)	
		DILI	+	65 (85.53)	11 (14.47)	0 (0)	0.680	141 (92.76)	11 (7.24)	0.436
			–	380 (82.07)	81 (17.49)	2 (0.43)		841 (90.82)	85 (9.18)	
		pulmonary infection	+	75 (80.65)	18 (19.35)	0 (0)	0.667	168 (90.32)	18 (9.68)	0.684
			–	370 (82.96)	74 (16.59)	2 (0.45)		814 (91.26)	78 (8.74)	
		hypoproteinemia	+	50 (89.29)	6 (10.71)	0 (0)	0.358	106 (94.64)	6 (5.36)	0.164
			–	395 (81.78)	86 (17.81)	2 (0.41)		876 (90.68)	90 (9.32)	
		leukopenia	+	28 (77.78)	8 (22.22)	0 (0)	0.654	64 (88.89)	8 (11.11)	0.496
			–	417 (82.90)	84 (16.70)	2 (0.40)		918 (91.25)	88 (8.75)	
		sputum smear-positive	+	134 (87.58)	19 (12.42)	0 (0)	0.101	287 (93.79)	19 (6.21)	**0.041**
			–	277 (79.83)	69 (19.88)	1 (0.29)		623 (89.77)	71 (10.23)	
rs1853259	A/G	fever	+	24 (33.33)	37 (51.39)	11 (15.28)	0.790	85 (59.03)	59 (40.97)	0.507
			–	145 (37.28)	192 (49.36)	52 (13.37)		482 (61.95)	296 (38.05)	
		drug resistance	+	26 (35.14)	38 (51.35)	10 (13.51)	0.948	90 (60.81)	58 (39.19)	0.852
			–	143 (36.95)	191 (49.35)	53 (13.70)		477 (61.63)	297 (38.37)	
		DILI	+	24 (35.29)	36 (52.94)	8 (11.76)	0.809	84 (61.76)	52 (38.24)	0.945
			–	145 (36.9)	193 (49.11)	55 (13.99)		483 (61.45)	303 (38.55)	
		pulmonary infection	+	29 (35.37)	40 (48.78)	13 (15.85)	0.814	98 (59.76)	66 (40.24)	0.613
			–	140 (36.94)	189 (49.87)	50 (13.19)		469 (61.87)	289 (38.13)	
		hypoproteinemia	+	15 (37.50)	21 (52.50)	4 (10.00)	0.775	51 (63.75)	29 (36.25)	0.665
			–	154 (36.58)	208 (49.41)	59 (14.01)		516 (61.28)	326 (38.72)	
		leukopenia	+	11 (36.67)	12 (40.00)	7 (23.33)	0.247	34 (56.67)	26 (43.33)	0.427
			–	158 (36.66)	217 (50.35)	56 (12.99)		533 (61.83)	329 (38.17)	
		sputum smear-positive	+	45 (35.71)	67 (53.17)	14 (11.11)	0.233	157 (62.30)	95 (37.70)	0.753
			–	114 (38.51)	134 (45.27)	48 (16.22)		362 (61.15)	230 (38.85)	

Bold value means P < 0.05.

### Haplotype analysis

The main haplotypes of *METTL3*, *METTL14*, and *WTAP* genes were detected *via* SHEsis software, and the frequency distributions of these haplotypes, including five for *METTL3* (AGC, ATC, GGC, GTA, GTC), four for *METTL14* (ATA, ATG, TCA, TTA), and three for *WTAP* (CA, CG, TA) are shown in **Table 3**. Compared with normal controls, the frequency of the *METTL3* AGC haplotype was significantly lower in PTB patients (*p* = 0.046), and the frequency of the *WTAP* TA haplotype was significantly higher (*p* = 0.035).

**Table 3:** Haplotype analysis of *METTL3*, *METTL14*, and *WTAP* genes in PTB patients and controls.

Haplotype	PTB [n (%)]	Controls [n (%)]	*P* value	*OR* (95% CI)
*METTL3* rs1139130-rs1061026-rs1061027				
AGC	27.94 (3.0)	45.14 (4.8)	**0.046**	0.615 (0.380,0.995)
ATC	573.06 (62.2)	551.86 (59.1)	0.177	1.137 (0.944,1.370)
GGC	47.91 (5.2)	41.62 (4.5)	0.457	1.175 (0.768,1.799)
GTA	171.85 (18.6)	184.76 (19.8)	0.531	0.929 (0.737,1.170)
GTC	101.09 (11.0)	110.38 (11.8)	0.562	0.919 (0.690,1.224)
*METTL14* rs1064034-rs4834698-rs62328061				
ATA	124.00 (13.4)	136.67 (14.6)	0.476	0.909 (0.699,1.181)
ATG	150.00 (16.3)	152.82 (16.4)	0.947	0.992 (0.775,1.182)
TCA	441.00 (47.8)	442.96 (47.4)	0.882	1.014 (0.845,1.217)
TTA	207.00 (22.5)	200.86 (21.5)	0.633	1.055 (0.847,1.314)
*WTAP* rs11752345- rs1853259				
CA	483.83 (52.5)	506.05 (54.2)	0.489	0.938 (0.781,1.125)
CG	353.17 (38.3)	367.95 (39.4)	0.454	0.958 (0.758,1.155)
TA	83.17 (9.0)	59.95 (6.4)	**0.035**	1.449 (1.026,2.046)

frequency < 0.03 in both controls & PTB patients has been dropped.

Bold value means P < 0.05.

### m6A methyltransferase transcription levels in the PTB group and the control group

**Figure 1:**
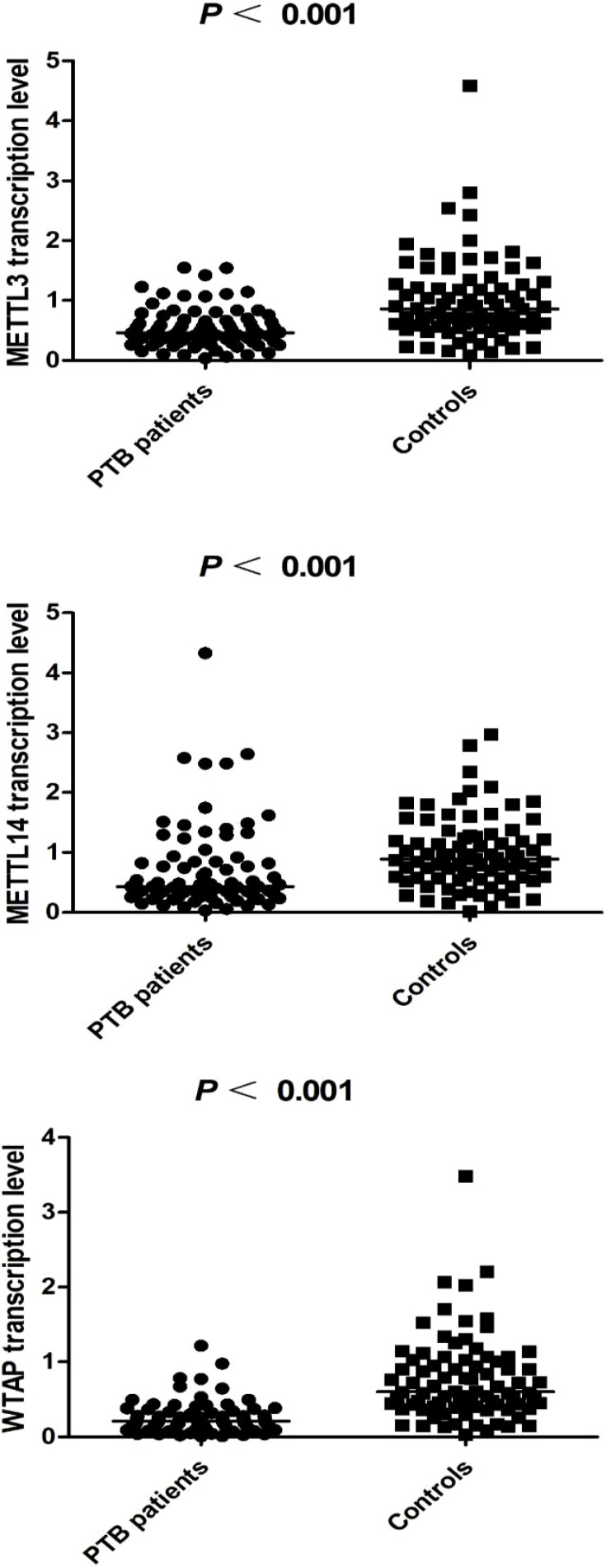
The transcription levels of METTL3, METTL14 and WTAP in PTB patients and controls.

The transcription levels of *METTL3*, *METTL14*, and *WTAP* in PBMCs were significantly lower in PTB patients than in normal controls (all *p* < 0.001) **(** **Figure 1** **)**. Associations between METTL3, METTL14, and WTAP mRNA levels and multiple clinical features were investigated in PTB patients. WTAP transcription level was significantly greater in PTB patients with drug resistance than in PTB patients without drug resistance (*p* = 0.030) (**Table 4**). *METTL3* transcription level was negatively associated with ESR (*p* = 0.035) and AST (*p* = 0.022) in PTB patients, and *METTL14* transcription level was negatively associated with ALT (*p* = 0.049) and AST (*p* = 0.004) (**Table 5**).

**Table 4:** Association between METTL3, METTL14, and WTAP transcription levels and several clinical features in PTB patients.

Group	+/-	N	METTL3 level	*P* value	METTL14 level	*P* value	WTAP level	*P* value
fever	+	15	0.361 (0.256,0.835)	0.644	0.400 (0.221,0.819)	0.490	0.158 (0.091,0.258)	0.226
	–	63	0.491 (0.307,0.682)		0.432 (0.292,0.936)		0.225 (0.125,0.344)	
drug resistance	+	5	0.639 (0.241,1.344)	0.333	0.936 (0.203,2.613)	0.364	0.384 (0.232,0.945)	**0.030**
	–	73	0.454 (0.307,0.674)		0.430 (0.280,0.831)		0.192 (0.102,0.309)	
DILI	+	8	0.371 (0.267,0.717)	0.521	0.648 (0.326,0.902)	0.489	0.237 (0.040,0.385)	0.895
	–	70	0.481 (0.307,0.686)		0.428 (0.271,0.865)		0.199 (0.120,0.336)	
pulmonary infection	+	11	0.342 (0.307,0.639)	0.651	0.363 (0.264,0.921)	0.937	0.170 (0.077,0.242)	0.220
	–	67	0.491 (0.306,0.700)		0.432 (0.287,0.845)		0.209 (0.125,0.374)	
hypoproteinemia	+	16	0.333 (0.271,0.609)	0.173	0.422 (0.255,1.014)	0.853	0.179 (0.048,0.278)	0.282
	–	62	0.495 (0.312,0.747)		0.431 (0.293,0.827)		0.217 (0.048,0.278)	
leukopenia	+	6	0.420 (0.150,0.847)	0.666	0.474 (0.166,1.251)	0.680	0.308 (0.141,0.565)	0.277
	–	72	0.462 (0.309,0.678)		0.431 (0.288,0.844)		0.199 (0.103,0.313)	
sputum smear-positive	+	27	0.437 (0.307,0.656)	0.781	0.515 (.264,0.937)	0.498	0.170 (0.097,0.284)	0.498
	–	51	0.495 (0.306,0.760)		0.430 (0.287,0.842)		0.209 (0.149,0.369)	

+/-: with/without; median (interquartile range); ^a^part of the study subjects of data missing. Bold value means P < 0.05.

**Table 5:** The correlation between METTL3, METTL14, and WTAP transcription levels and ESR, TBIL, ALT, AST of PTB patients.

Clinical parameters	METTL3 level		METTL14 level		WTAP level	
	*r_s_*	*P* value	*r_s_*	*P* value	*r_s_*	*P* value
ESR	-0.243	**0.035**	-0.054	0.642	-0.034	0.772
TBIL	0.131	0.260	0.057	0.626	-0.018	0.878
ALT	-0.079	0.499	-0.227	**0.049**	-0.083	0.476
AST	-0.264	**0.022**	-0.331	**0.004**	-0.113	0.336

r_s_:Spearman’s rank correlation coefficient. Bold value means P < 0.05.

### Associations between m6A methyltransferase gene polymorphisms and their transcription levels in PTB patients

The Associations between genes variation in m6A methyltransferase METTL3, METTL14, and WTAP and their transcription levels were assessed in 64 PTB patients, and no significant differences were detected (all *p* > 0.05) (**Table 6**).

**Table 6:** Association between *METTL3*, *METTL14*, and *WTAP* genes polymorphisms with their transcription levels in PTB patients.

METTL3 SNP	Genotype	number	METTL3 level	P value
rs1139130	AA	32	0.554 (0.279,0.678)	0.325
	AG	27	0.409 (0.306,0.529)	
	GG	5	0.559 (0.431,0.760)	
rs1061026	TT	55	0.470 (0.312,0.665)	0.412
	TG	9	0.495 (0.165,0.642)	
	GG	0	–	
rs1061027	CC	42	0.474 (0.307,0.669)	0.981
	CA	20	0.490 (0.303,0.684)	
	AA	2	0.436 (0.314,0.559)	
*METTL14* SNP	Genotype	number	METTL14 level	*P* value
rs1064034	TT	32	0.423 (0.261,1.137)	0.917
	AT	26	0.422 (0.249,0.826)	
	AA	6	0.404 (0.253,1.879)	
rs62328061	AA	47	0.364 (0.255,0.819)	0.153
	AG	14	0.660 (0.359,1.162)	
	GG	3	0.489 (0.319,1.625)	
rs4834698	TT	16	0.449 (0.330, 0.811)	0.690
	TC	37	0.400 (0.256, 0.831)	
	CC	11	0.343 (0.111,1.397)	
*WTAP* **SNP**	Genotype	number	WTAP level	*P* **value**
rs11752345	CC	52	0.207 (0.103,0.329)	0.618
	CT	12	0.178 (0.082,0.315)	
	TT	0	–	
rs1853259	AA	27	0.225 (0.068,0.344)	0.635
	AG	29	0.185 (0.099,0.328)	
	GG	8	0.246 (0.165,0.307)	

Median (interquartile range).

## Discussion

Many novel genetic variants associated with susceptibility to PTB have been identified *via* genome-wide association studies and candidate gene studies, but unearthing the full range of PTB susceptibility variations remains a challenge. Given the important role of m6A modification in the development of multiple diseases, potential associations between genetic variation of m6A-modified genes and disease susceptibility have also attracted increased attention in recent years (27, 28). The classic m6A methyltransferase complex mainly consists of METTL3, METTL14, and WTAP, which mediate m6A methylation of mRNA. METTL3 is a vital methyltransferase as an S-adenosylmethionine-binding subunit, whereas METTL14 is an RNA-binding scaffold for substrate recognition. WTAP interacts with METTL3 and METTL14, then localizes them into nuclear speckles (29, 30). Notably the SNPs located in these m6A methyltransferases are reportedly associated with disease susceptibility *via* m6A methylation and related biological processes (19, 20, 31). To the best of our knowledge, the current study is the first to investigate associations between polymorphic variants of the m6A methyltransferase genes *METTL3*, *METTL14*, and *WTAP* and PTB susceptibility. The study provides strong evidence that the *METTL14* gene variant rs62328061 and the *WTAP* gene variant rs11752345 are associated with the risk of PTB, and that METTL3, METTL14, and WTAP levels are decreased in PTB patients.

To date, studies on genetic variation of *METTL3* and disease susceptibility have mainly focused on cancers. Lin et al. (32) suggested that the combination of rs1139130, rs1263801, rs1061026, and rs1061027 variants in the *METTL3* gene could reduce the risk of Wilms’ tumor in children. Another study reported that these four SNPs were also related to higher susceptibility to neuroblastoma (19). In contrast with these findings, rs1061027, rs1139130, and rs1061026 variants were not significantly associated with PTB susceptibility in the present study. Compared with single SNPs, association studies based on haplotypes of multiple markers can significantly enhance the mapping and characterization of disease susceptibility genes (33). In this study, we assessed whether the main haplotypes consisting of the aforementioned three polymorphisms were associated with PTB, and the AGC haplotype was significantly associated with a lower risk of PTB. These results suggest that there may be weak associations between *METTL3* gene variation of and PTB susceptibility, and these variants may interact with each other to modify the PTB risk. This should be confirmed in future studies with larger sample sizes. In addition, rs1139130 polymorphism was also significantly associated with drug resistance and DILI in PTB patients. This further proved that *METTL3* gene variation was involved in the pathogenesis of PTB, and indicated that this SNP could be used to predict adverse reactions in patients, thus contributing to the formulation of more appropriate treatment measures.

In previous studies, *METTL14* gene rs62328061 polymorphism was significantly associated with reduced susceptibility to neuroblastoma (34), and rs1064034, rs298982 polymorphisms were significantly associated with reduced susceptibility to Wilms’ tumor (21). In the current study, the rs62328061 GG genotype was associated with an increased risk of PTB, but there was no significant association between the rs1064034 variant and PTB susceptibility. These results provided key indications of an association between *METTL14* gene variation and PTB susceptibility. In PTB patients, a decreased frequency of the rs62328061 G allele was associated with leukopenia, and an increased frequency of the rs4834698 TC genotype was associated with hypoproteinemia. This confirmed the important role of *METTL14* gene variation in the development of PTB, and further functional studies are needed to investigate the molecular mechanisms involved in the effects of rs62328061 on PTB risk. The present study also investigated associations between *WTAP* gene polymorphisms and the risk of PTB, and the *WTAP* rs11752345 CT genotype and T allele frequencies were significantly increased in PTB patients. Moreover, the rs11752345 T allele was closely related to sputum smear in PTB patients. These results suggested that the CT genotype/T allele of rs11752345 may increase the risk of PTB, and the T allele may be useful for distinguishing different phenotypes in PTB patients.

Increasing studies indicate that transcription levels of m6A methyltransferases are closely related to the pathogenesis and progression of many diseases. Dysregulation of METTL3 is considered an important factor affecting the progression of various malignant tumors such as endometrial cancer (35) and bladder cancer (36). Another study detected lower METTL14 expression in colorectal cancer (37). Functional m6A methyltransferase SNPs may affect m6A methyltransferase expression and thus modify disease susceptibility. Hence, we designed a case-control study to determine METTL3, METTL14, and WTAP transcription levels in PTB patients to investigate the possible mechanism of SNP-mediated PTB susceptibility. Transcription levels of *METTL3*, *METTL14*, and *WTAP* were significantly decreased in PTB patients compared to controls. This suggested that METTL3, METTL14, and WTAP may be involved in PTB occurrence, and their levels may be used as auxiliary indicators for PTB diagnosis. This must be verified in the future by well-designed studies. WTAP level was also significantly associated with drug resistance, ALT, and AST, and METTL3 level was negatively associated with ESR and AST in PTB patients. These findings contribute to improving our understanding of the role of m6A methyltransferases in PTB development. Lastly, we analyzed potential associations between m6A methyltransferase gene variants and their transcription levels in PTB patients. No significant associations were detected, possibly due to the small sample size.

The major strengths of this study include its novelty and rational design, but the study also had several limitations. It was focused on the analysis of genetic factors in PTB, but it did not assess the effects of environmental factors on PTB, or interactions between environmental factors and genetic variation. Second, associations between gene variation and the prognosis of PTB were not analyzed. Lastly, the study lacked a functional investigation into the effects of m6A methyltransferase on PTB pathogenesis.

In summary, the current study indicates that polymorphisms of *METTL14* rs62328061 and *WTAP* rs11752345 may contribute to increased PTB susceptibility, and several SNPs in *METTL3*, *METTL14*, and *WTAP* genes were associated with the clinical features including drug resistance, DILI, and leukopenia in PTB patients. The study demonstrated the important roles of alterations in METTL3, METTL14, and WTAP in PTB, and these m6A methyltransferases in the development of PTB. In order to further validate these findings and determine the underlying biological mechanisms involving these m6A methyltransferases in PTB, more studies incorporating larger sample sizes and different ethnic populations are warranted.

## Data availability statement

The data presented in the study are deposited in the dbSNP (1063446) . Further inquiries can be directed to the corresponding authors.

## Ethics statement

This study was approved by the Ethical Committee of Anhui Medical University. The patients/participants provided their written informed consent to participate in this study.

## Author contributions

T-PZ and H-ML designed the study. H-ML conducted the experiment. RL performed the statistical analyses. L-JW and QH participated in the collection of samples. H-ML and T-PZ drafted the manuscript. All author contributed to manuscript revision. All the authors approved the final submitted version.
